# Eye signs as a novel risk predictor in pulmonary arterial hypertension associated with systemic lupus erythematosus

**DOI:** 10.1186/s42358-024-00356-0

**Published:** 2024-02-29

**Authors:** Jianbin Li, Jiangbiao Xiong, Pengcheng Liu, Yilin Peng, Shuang Cai, Xia Fang, Shujiao Yu, Jun Zhao, Rui Wu

**Affiliations:** Department of Rheumatology and Immunology, The First Affiliated Hospital, Jiangxi Medical College, Nanchang University, No. 17 Yongwaizheng Street, Donghu District, Nanchang City, Jiangxi Province 330006 China

**Keywords:** Systemic lupus erythematosus, Pulmonary arterial hypertension, Conjunctival vasculature, Eye sign, Vascular density, Microvascular flow index

## Abstract

**Objective:**

To investigate the role of eye signs in predicting poor outcomes in systemic lupus erythematosus (SLE) patients with pulmonary arterial hypertension (PAH).

**Methods:**

This prospective observational study recruited patients diagnosed with SLE-PAH from Jan. 2021 to Dec. 2021 at the First Affiliated Hospital of Nanchang University; those with other potential causes of PAH were excluded. The evaluation of various parameters, such as N-terminal prohormone of brain natriuretic peptide (NT-proBNP), 6-minute walking distance (6MWD), World Health Organization functional class (WHO-FC), echocardiography, and risk stratification based on the 2015 European Society of Cardiology (ESC)/European Respiratory Society (ERS) Guidelines, was conducted at intervals of every 1–3 months, and a 6-month follow-up period was observed. The primary outcome measure considered improvement if there was a decline in the risk stratification grade at the end point and unimproved if there was no decline. Conjunctival microvascular images were observed and recorded.

**Results:**

A total of 29 SLE-PAH patients were enrolled, comprising 12 in the improved group and 17 in the nonimproved group. All SLE-PAH patients showed various manifestations of eye signs, including vessel twisting, dilation, ischaemic areas, haemorrhages, reticulum deformity, and wound spots. The nonimproved group exhibited significantly lower vessel density (VD) and microvascular flow index (MFI) of conjunctival microvascular images than the improved group. Correlation analysis revealed that VD displayed a negative correlation with the WHO-FC (*r* = −0.413, *p* = 0.026) and NT-proBNP (*r* = −0.472, *p* = 0.010), as well as a positive correlation with the 6MWD (*r* = 0.561, *p* = 0.002). Similarly, MFI exhibited a negative correlation with WHO-FC (*r* = −0.408, *p* = 0.028) and NT-proBNP (*r* = −0.472, *p* = 0.010) and a positive correlation with 6MWD (*r* = 0.157, *p* = 0.004). Multivariate logistic regression analysis indicated that VD (OR 10.11, 95% CI 1.95–52.36), MFI (OR 7.85, 95% CI 1.73–35.67), NT-proBNP, and 6MWD were influential factors in predicting the prognostic improvement of SLE-PAH patients. ROC curve analysis demonstrated that VD, MFI, 6MWD, and NT-proBNP (with respective AUC values of 0.83, 0.83, 0.76, and 0.90, respectively) possessed a sensitivity and specificity of 75 and 100%, as well as 83 and 100%, respectively. Regarding prognostic prediction, VD and MFI exhibited higher sensitivity than 6MWD, whereas MFI displayed higher sensitivity and specificity than NT-proBNP.

**Conclusion:**

SLE-PAH can lead to various conjunctival microvascular manifestations in which vascular density and microvascular flow index can be used to assess cardiopulmonary function and predict therapeutic efficacy and prognosis in SLE-PAH patients.

## Introduction

Systemic lupus erythematosus (SLE) is an autoimmune disease that affects multiple organs and systems in the human body. Pulmonary arterial hypertension (PAH) is one of the most severe complications of SLE and is associated with poor prognosis and high mortality rates. The 5-year survival rate of SLE-related PAH ranges from 50 to 62.9% [1, 2]. PAH can lead to systemic microcirculatory impairments [3]. Microcirculation refers to the circulation of blood between arterioles and venules, comprising the peripheral component of the circulatory system. It serves as a crucial site for substance exchange between blood and tissue cells. Common sites for assessing systemic microcirculation include the nail bed, tongue, and conjunctiva. Compared to other sites for evaluating systemic microcirculation, the conjunctiva is minimally influenced by external temperature and offers closer insights into visceral microcirculatory alterations [4].

**Fig. 1:**
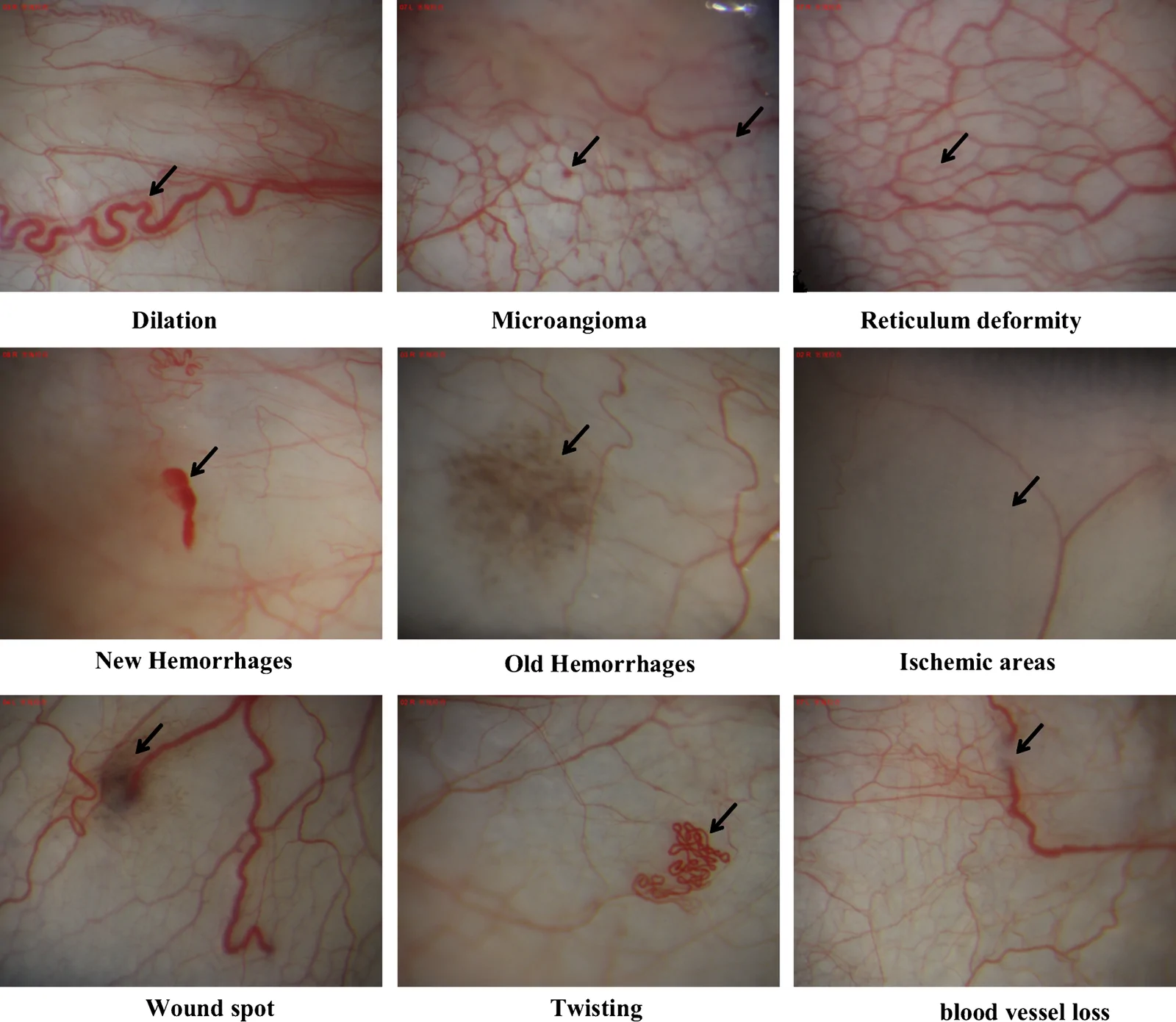
Featured microvascular alterations of eye signs: ischaemic area (more than 3 capillary grid areas without vessels under a 40× microscope); reticulum deformity (the capillaries increase, the mesh decreases, and the dendrites become grid-like); microangioma (can be divided into local round, fusiform, cystic dilation, or isolated and scattered around the vessel); and wound spot (vascular blind end-shaped brown, purple, or dark blue material deposition). Bleeding is exudative (fuzzy vessel wall), and there are ruptures (wound spots, patches) around capillaries

Eye signs, introduced by Professor Li Guoxian in 1988, are a reliable and effective method for evaluating the severity of blood stasis syndrome. Eye signs are known for their specificity and sensitivity in assessing the condition [5]. In clinical practice, we have observed some distinct manifestations of conjunctival microcirculation in patients with hypercoagulability. These included vessel twisting, dilation, haemorrhages, ischaemic areas, reticulum deformity, microangioma, and wound spots (Fig. 1), collectively referred to as eye signs. In large-scale, multicentre studies, eye signs have been proven to be a valuable diagnostic tool for assessing systemic hypercoagulability and microcirculation disorders. Furthermore, we established relevant scoring criteria to evaluate patients with microcirculatory disturbances [6]. It was preliminarily discovered that eye signs were associated with cardiopulmonary function and can be used to assess the risk of mortality in systemic lupus erythematosus with pulmonary arterial hypertension [7]. To further investigate the role of eye signs in pulmonary arterial hypertension and explore the predictive value of eye signs in the treatment response and prognosis of SLE-PAH, this prospective observational study observed conjunctival microvessel manifestations of eye signs and compared the differences in eye sign parameters between improved and nonimproved groups after 6 months of follow-up.

## Materials and methods

### Subjects

This prospective observational study consecutively recruited patients diagnosed with SLE-PAH at the First Affiliated Hospital of Nanchang University from January 2021 to December 2021. All enrolled patients met the 2019 European League Against Rheumatism/American College of Rheumatology classification criteria for systemic lupus erythematosus [8]. The diagnostic criteria for PAH included mean pulmonary arterial pressure (mPAP) > 20 mmHg, pulmonary artery wedge pressure (PAWP) ≤ 15 mmHg, and pulmonary vascular resistance (PVR) > 2 Wood units, as determined by right heart catheterization (RHC), or systolic pulmonary arterial pressure (sPAP) ≥ 40 mmHg, as assessed through transthoracic echocardiography combined with clinical manifestations [9]. Patients with interstitial lung disease, congenital heart disease, chronic obstructive pulmonary disease, pulmonary thromboembolism, or human immunodeficiency virus infection were excluded from this study. Additionally, patients presenting with conjunctival trauma, the presence of pterygium, inflammatory or allergic processes, and dry eyes, with or without Sjogren syndrome, were also excluded. This study adhered to the principles of the Declaration of Helsinki and was approved by the Ethics Committee of the First Affiliated Hospital of Nanchang University. Written informed consent was obtained from all patients.

### Study design

During this study, patients underwent regular visits every 1–3 months. Risk stratification based on the 2015 European Society of Cardiology (ESC)/European Respiratory Society (ERS) Guidelines was conducted [10], involving the evaluation of various parameters such as N-terminal prohormone of brain natriuretic peptide (NT-proBNP), 6-minute walking distance (6MWD), World Health Organization functional class (WHO-FC), and echocardiography. These assessments were carried out at intervals of 1–3 months over a 6-month follow-up period. The primary outcome of this study was the risk stratification of PAH, considering an improvement if there was a decline in the risk stratification grade at the endpoint and no improvement otherwise.

It is important to note that due to resource and technical limitations, our study was unable to include right heart catheterization (RHC). As a result, the diagnosis and risk stratification of PAH were primarily based on noninvasive methods, such as echocardiography and other clinical assessments. We recognize this as a limitation of our study and suggest that future research should consider including this diagnostic tool for a more comprehensive evaluation.

### Conjunctival microvasculature

An SLM-7E digital slit lamp was used to detect conjunctival microvasculature. Each subject rotated their eyeballs up, down, left, and right to fully expose the required observation range. Eight conjunctival microvascular images were observed and recorded when the binoculars were in four different positions. The observations included the following: vessel twisting, dilation, haemorrhages, ischaemic areas, reticulum deformity, microangioma, blood vessel loss and wound spots (Fig. 1). For the vessel density (VD) calculation method, the image under a 40× mirror was divided into 16 grids with three horizontal lines and three vertical lines, and the number of lines passing through the grid was calculated and averaged. For the microvascular flow index (MFI), the image under a 100× microscope was divided into four directions, and the average was taken after integration. Normal flow was counted as 3 points, sluggish flow was 2 points, intermittent flow was 1 point, and no flow for at least 20 s was 0 points [11].

### Statistical analysis

SPSS version 26.0 (IBM, Armonk, NY, USA) was used for statistical analysis. Descriptive statistics are reported for continuous variables as the means ± standard deviations for normally distributed data or as the medians (interquartile ranges) for nonnormally distributed data. Categorical variables are presented as frequencies and percentages. Comparisons between two groups were assessed using either the independent samples t test or the Mann‒Whitney U test for continuous variables and the chi-square test or Fisher’s exact test for categorical variables. Correlation analysis was conducted using Pearson’s test or, if appropriate, Spearman’s test. Logistic regression analysis was employed to identify potential risk factors and generate a receiver operating characteristic (ROC) curve. The discriminatory performance of potential predictive factors for SLE-PAH was evaluated by calculating the area under the curve (AUC), sensitivity, and specificity. The cut-off point for identifying potential predictive factors was determined through ROC analysis. All statistical tests were two-tailed, and statistical significance was defined as *p* < 0.05.

## Results

### Patient characteristics at baseline in the improved and nonimproved groups

According to Table 1, there were significant differences (*p* < 0.05) in the clinical parameters, including cardiac functional classification, NT-proBNP levels, 6MWD, conjunctival ischaemic areas, reticulum deformity, VD, and MFI, between the improved and nonimproved groups of SLE-PAH patients. Both groups of SLE-PAH patients presented with various conjunctival manifestations, such as vessel twisting, dilation, ischaemic areas, haemorrhages, reticulum deformity, and wound spots. Among these, conjunctival microangioma, vessel twisting, and wound spots were the most frequently observed changes.

**Table 1:** General characteristics and eye sign presentation of the two patient groups

	Improved group (n = 12)	Non-improved group (n = 17)	*P*
Gender (F/M)	12 (100%)	16 (94.1%)/1(5.9%)	0.393
Age (years)	41.42 ± 10.10	38.41 ± 12.19	0.565
Functional level			0.006^**^
I	6 (50%)	0	
II	4 (33.33%)	2 (11.76%)	
III–IV	2 (16.67)	10 (58.82%)	
NTproBNP	297 (135, 586)	1200 (688, 2155)	0.000*
6MWD (m)	420 (400, 470)	360 (300, 410)	0.018*
Medication			
Corticosteroids	12 (100%)	17 (100%)	
Endothelin inhibitors	4 (33.3%)	8 (47.1%)	0.460
PDE5 inhibitors	2 (16.7%)	3 (17.6%)	0.945
Combination therapy	6 (50%)	6 (35.3%)	0.428
eye signs			
Ischemic areas, n (%)	6 (50%)	8 (47.1%)	0.876
Reticulum deformity, n (%)	4 (33.3%)	6 (35.3%)	0.913
Microangioma, n (%)	6 (50%)	9 (52.9%)	0.876
Twisting, n (%)	11 (91.7%)	16 (94.7%)	0.798
Dilation, n (%)	7 (58.3%)	5 (29.4%)	0.119
Wound spot, n (%)	5 (41.7%)	13 (76.5%)	0.057
Hemorrhage, n (%)	3 (25%)	5 (29.4%)	0.793
blood vessel loss, n (%)	2 (16.7%)	5 (29.4%)	0.430
VD (n/mm2)	1.83 ± 0.79	0.98 ± 0.42	0.001^**^
MFI	2.4 (2.0, 2.87)	1.2 (1.0, 1.35)	0.002^**^

*6MWD* 6-minute walking distance; *WHO FC* World Health Organization functional class; *NT-proBNP* N-terminal prohormone of brain natriuretic peptide; *VD* vessel density; *MFI* microvascular flow index; *P < 0.05, **P < 0.01

### The correlation between eye sign indicators and cardiopulmonary function

**Fig. 2:**
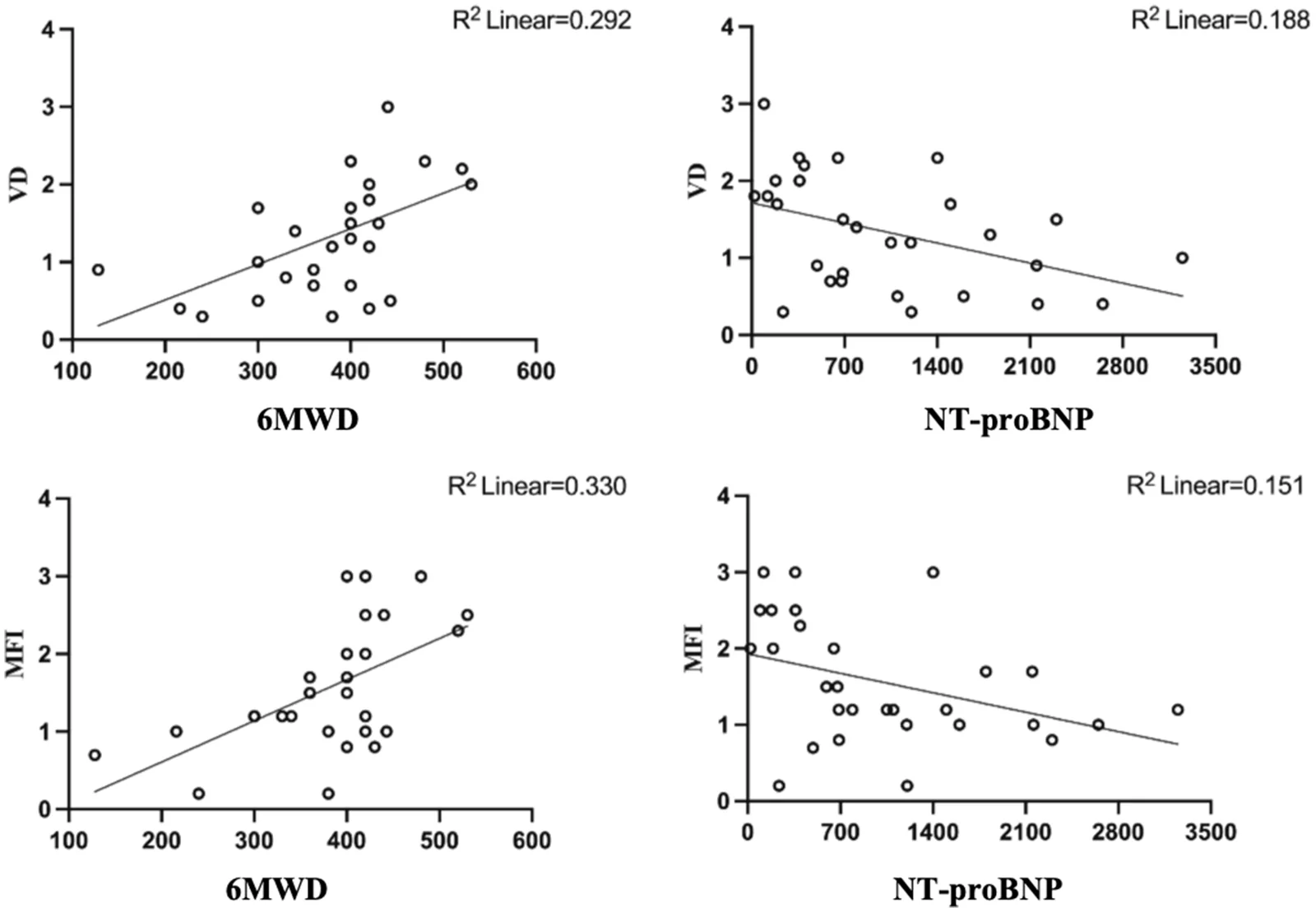
Scatter plot of the correlation analysis of vessel density (VD), microvascular flow index (MFI), 6-minute walking distance (6MWD) and N-terminal prohormone of brain natriuretic peptide (NT-proBNP)

Spearman correlation analysis showed that VD and MFI were negatively correlated with NT-proBNP and WHO-FC (*r* < 0, *P* < 0.05) and positively correlated with 6MWD (*r* > 0, *P* < 0.05) (Table 2, Fig. 2).

**Table 2:** Correlation analysis of eye sign indicators and parameters of SLE-PAH

Parameters	6MWD		WHO-FC		NT-proBNP	
	*r*	*P*	*r*	*P*	*r*	*P*
VD	0.561**	0.002	−0.413*	0.026	−0.472*	0.010
MFI	0.517**	0.004	−0.408*	0.028	−0.492**	0.007

*6MWD* 6-minute walking distance; *WHO FC* World Health Organization functional class; *NT-proBNP* N-terminal prohormone of brain natriuretic peptide; *VD* vessel density; *MFI* microvascular flow index; *P < 0.05, **P < 0.01

### Comparison of various parameters in different PAH risk stratifications

Based on the 2015 European guidelines, all patients were divided into low-risk, moderate-risk, and high-risk groups using a multiparameter risk stratification scale, as shown in Table 3. NT-proBNP, 6MWD, VD, and MFI showed significant differences among different risk groups, while pulmonary arterial pressure (PH) did not show significant differences.

**Table 3:** Comparison of various parameters among the three PAH risk-stratified groups

GROUP	Low risk (n = 8)	Moderate risk (n = 14)	High risk (n = 7)	*P* value
PH (mmHg)	44.38 ± 3.89	47.86 ± 9.49	54.71 ± 23.91	0.336
NT- proBNP (pg/ml)	251.35 ± 205.83	1059.49 ± 643.79	1865.44 ± 887.56	0.001**
6MWD (m)	443.75 ± 51.81	391.64 ± 47.93	277.71 ± 91.62	0.001**
VD (n/mm2)	2.1 ± 0.42	1.08 ± 0.65	0.96 ± 0.52	0.001**
MFI	2.35 ± 0.35	1.39 ± 0.77	0.97 ± 0.47	0.001**
Eye sign				

*6MWD* 6-minute walking distance; *NT-proBNP* N-terminal prohormone of brain natriuretic peptide; *VD* vessel density; *MFI* microvascular flow index; *P < 0.05, **P < 0.01

### Assessment of risk parameters in predicting treatment response and prognosis of SLE-PAH

In univariable logistic analysis, variables such as VD, MFI, WHO-FC, 6MWD, and NT-proBNP demonstrated statistical significance in both the improved and nonimproved groups (*p* < 0.05). In the multivariable analysis, age was included as a confounding factor. In Model 1 adjusted for VD, VD emerged as a significant predictor of SLE-PAH (HR 10.11, 95% CI 1.95–52.36, *p* = 0.006). Likewise, in Model 2 adjusted for MFI, MFI remained a significant predictor (HR 7.85, 95% CI 1.73–35.67, *p* = 0.008). Similarly, in the other adjusted models, WHO-FC (HR 0.12, 95% CI 0.02–0.75, *p* = 0.024), 6MWD (HR 1.02, 95% CI 1.00–1.03, *p* = 0.039), and NT-proBNP (HR 0.99, 95% CI 0.99–1.00, *p* = 0.010) were also identified as significant predictors (Table 4).

**Table 4:** Performance of potential predictive factors with cut-off points in adjusted univariate and multivariate logistic regression models for SLE-PAH

	Univariate		Multivariate		
	OR (95% CI)	*P* value	OR (95% CI)	*P* value	
VD	9.69 (1.86–50.36)	0.007	10.11 (1.95–52.36)	0.006**	
MFI	7.74 (1.74–34.52)	0.007	7.85 (1.73–35.67)	0.008**	
WHO-FC	0.16 (0.04–0.69)	0.009	0.12 (0.02–0.75)	0.024*	
Age	1.02 (0.96–1.09)	0.475			
6MWD	1.02 (1.00–1.03)	0.047	1.02 (1.00–1.03)	0.039*	
NT-proBNP	0.99 (0.99–1.00)	0.011	0.99 (0.99–1.00)	0.010*	

*6MWD* 6-minute walking distance; *WHO FC* World Health Organization functional class; *NT-proBNP* N-terminal prohormone of brain natriuretic peptide; *VD* vessel density; *MFI* microvascular flow index; *P < 0.05, **P < 0.01

ROC curve analysis demonstrated that VD had a threshold of 1.75 (AUC 0.83, 95% CI 0.62–1.00, sensitivity 75%, specificity 100%), MFI had a threshold of 1.85 (AUC 0.83, 95% CI 0.62–1.00, sensitivity 83%, specificity 100%), 6MWD had a threshold of 461.5 (AUC 0.76, 95% CI 0.58–0.94, sensitivity 25%, specificity 100%), and NT-proBNP had a threshold of 664.25 (AUC 0.90, 95% CI 0.78–1.00, sensitivity 88%, specificity 83%). All these indicators effectively differentiated between SLE-PAH patients with improvement and those without improvement (*P* < 0.001, *P* < 0.001, *P* < 0.001, *P* < 0.001, *P* < 0.001, respectively) (Table 4).

## Discussion

Various microvascular changes in eye signs were observed in SLE-PAH patients, including decreased vessel number (ischaemic areas), increased vessel number (reticulum deformity), changes in vascular morphology (twisting, dilation, microangioma) and vascular venous injury (haemorrhage and wound spots). Decreased VD and MFI were associated with poor cardiopulmonary function. Compared to conventional risk assessment indicators for pulmonary hypertension, WHO functional class (HR 0.12, 95% CI 0.02–0.75, *p* = 0.024), 6MWD (HR 1.02, 95% CI 1.00–1.03, *p* = 0.039), NT-proBNP (HR 0.99, *p* = 0.010), VD (HR 10.11, *p* = 0.006) and MFI (HR 7.85, *p* = 0.008) appeared to be more effective in predicting inadequate therapeutic response and poor prognosis of SLE-PAH.

PAH is a severe and progressive pulmonary vascular disease characterized by elevated pulmonary artery pressure and increased pulmonary artery resistance, leading to right ventricular failure and mortality. The REVEAL registry study in the United States revealed that CTD-related PAH accounts for 25.3% of all PAH cases. SLE and systemic sclerosis (SSc) are the most common CTDs associated with PAH [12]. An analysis of the causes of death among SLE patients in China over the past 30 years found that SLE-PAH was the third leading cause of death among SLE patients [13]. The pathogenesis of CTD-PAH is complex, making treatment challenging, as even with novel targeted combination therapies, the three-year mortality rate among moderate- to high-risk patients remains as high as 56% [14].

Assessing the risk of pulmonary arterial hypertension (PAH) is crucial in guiding standardized treatment and reducing mortality rates among PAH patients. Therefore, PAH risk assessment methods were introduced in the 2012 REVEAL study, the 2015 European PAH guidelines, and the 2018 6th World Symposium on Pulmonary Hypertension (WSPH) [10, 15, 16]. Evaluation indicators include clinical manifestations, the level of cardiac function, plasma brain natriuretic peptide (BNP) levels, cardiac echocardiography and haemodynamic levels. However, in these risk stratifications, some risk parameters require high measurement conditions, such as cardiac index (CI) and mixed venous oxygen saturation (SvO2) derived by right heart catheterization, limiting their practicality. Research has been conducted to identify simpler risk assessment indicators for daily clinical practice, such as age, right atrial area, pulmonary arteriole diameter, serum iron levels, red cell distribution width, and blood uric acid levels [17–19]. However, the predictive value of some risk assessment indicators used in CTD-PAH remains controversial. Current studies, particularly those involving nailfold video capillaroscopy [20], have revealed the importance of microvascular damage in systemic sclerosis (SSc)-associated PAH and support the hypothesis of systemic microvascular involvement in idiopathic PAH. These findings further suggest that although our research primarily focused on SLE and CTD-PAH, microvascular damage and its related clinical manifestations may also be equally important in other types of PAH.

Vessel density (VD) and microvascular flow index (MFI) are essential parameters in microcirculation [21]. VD refers to the number of blood vessels in a unit area. The level of vascular density reflects the distribution of microvessels and the degree of blood supply. The formation and regulation of vascular density involve multiple mechanisms, including angiogenesis, proinflammatory cytokine release, and vascular constriction and dilation. For instance, angiogenic factors such as VEGF stimulate the formation of new blood vessels. The release of cytokines and chemical mediators may induce vascular constriction or dilation, thereby regulating vascular density. MFI assesses the haemodynamic characteristics of microcirculation by measuring the average flow velocity and density of red blood cells in microvessels. It reflects the blood flow velocity and volume in the microcirculation. Clinically, changes in MFI effectively indicate alterations in blood perfusion. Higher MFI values indicate faster blood flow velocity and larger vascular blood volume [7].

VD and MFI have gained increasing attention as crucial assessments for haemodynamic changes and systemic microcirculation disorders in critically ill patients. Research on microcirculation evaluation in sepsis and critical care monitoring has shown that VD and MFI can determine the fluid resuscitation needs of ICU patients [22, 23]. There is currently a lack of relevant research on VD and MFI for assessing SLE-PAH. A previous cross-sectional study showed that VD and MFI in conjunctival microvasculature were associated with risk levels of mortality in SLE-PAH [7]. Further investigation into the measurement of VD and MFI in SLE-PAH can contribute to evaluating the systemic haemodynamic changes caused by PAH and a better understanding of the pathophysiological mechanisms of SLE-PAH.

The conjunctival microcirculation, as a crucial window into systemic microcirculation, indirectly reflects the overall state of microcirculation through the observation and measurement of conjunctival vascular density, vessel diameter, blood flow velocity, vascular branching pattern, endothelial wall, vascular reactivity and perfusion status [24]. The normal course of conjunctival microcirculation involves arterioles leading to capillaries and then proceeding through venules. Arterioles have a relatively straight course, while venules exhibit slight curvature. The calibres of both arteries and veins are uniform, with an arterial/venous ratio of approximately 1:2. Capillaries form a branching network. Under normal conditions, blood flow dynamics in arterioles appear as linear flow, while venules exhibit linear or granular flow. Capillaries show granular flow with occasional mild red blood cell aggregation. In comparison with other common methods for sublingual microcirculation and nailfold microcirculation, conjunctival microcirculation offers advantages such as being unaffected by external temperature and having a stronger correlation with visceral blood vessels [21, 25]. However, it also has certain limitations, including difficulties in cooperation during testing, complexity in examination procedures, and greater requirements for equipment and personnel expertise. To solve complex operational problems, eye signs were assessed through a clinical observational study involving a large sample size. Focusing on the distinct manifestations of conjunctival microcirculation changes in patients with a hypercoagulable state, a conjunctival vascular panel was defined, including vessel twisting, dilation, haemorrhages, ischaemic areas, reticulum deformity, microangioma, and wound spots (Fig. 1). This set of distinctive features is referred to as “eye signs.” Compared to conjunctival microcirculation examination, the detection of eye signs is simpler, as they can be observed using a conventional slit lamp or even with the naked eye. Clinical validation of eye signs demonstrates their high accuracy in assessing thrombosis or hypercoagulability, exhibiting a strong correspondence with nailfold capillary microcirculation [26, 27].

In patients with PAH, elevated pulmonary arterial pressure leads to increased pulmonary vascular resistance. This results in decreased blood flow speed and capacity in pulmonary capillaries, causing a reduction in pulmonary blood volume and oxygen supply [28]. Consequently, there is a decline in oxygenation function and impairment of cardiac performance, which are caused by the severe condition of PAH and extremely elevated vascular resistance. Therefore, in cases of PAH occurring in SLE, bulbar conjunctival microvessels can exhibit widespread changes, such as twisting, haemorrhages, dilation, and microangioma. However, more importantly, in this study among patients with SLE-PAH who had a poor prognosis after treatment, microcirculatory VD and MFI were significantly reduced. Thus, clinically, for patients with significant reductions in VD and MFI, intensified treatment is necessary. Additionally, close monitoring of the trends in VD and MFI can provide an important basis for assessing treatment efficacy and predicting prognosis. This is very important for guiding clinical practice in SLE-PAH.

This study was conducted at a single centre and involved a relatively small sample size. Moreover, it included only patients from China, which may limit the generalizability of the findings to other populations. To confirm the clinical significance of vessel density (VD) and the microvascular flow index (MFI) in pulmonary arterial hypertension (PAH), further research with more diverse and larger sample sizes is essential. While additional clinical trials are necessary to refine parameter calculations and achieve standardization, the undeniable convenience and feasibility of conjunctival microcirculation assessment make it a promising method that warrants further clinical promotion and application in various demographic settings.

## Data Availability

Primary data are available upon the corresponding author request.
